# Risk of breast cancer among users of aspirin and other anti-inflammatory drugs

**DOI:** 10.1038/sj.bjc.6602003

**Published:** 2004-06-29

**Authors:** L A García Rodríguez, A González-Pérez

**Affiliations:** 1 Centro Español de Investigación Farmacoepidemiológica (CEIFE), C/Almirante 28 2°, 28004 Madrid, Spain

**Keywords:** antiinflammatory drug, aspirin, steroids, epidemiology

## Abstract

We conducted a cohort study with a nested case–control analysis to evaluate the effect of anti-inflammatory drugs in breast cancer incidence using the General Practice Research Database. Women taking aspirin and paracetamol for 1 year or longer had an odds ratio (OR) of 0.77 (95 percent confidence interval (95% CI): 0.62,0.95) and 0.76 (95% CI: 0.65,0.88), respectively, compared to nonusers. Daily doses of aspirin (75 mg) and paracetamol (up to 2000 mg) showed the greatest reduced risk. Use of non-aspirin nonsteroidal anti-inflammatory drugs for more than 1 year was not associated with a reduced risk of breast cancer (OR=1.00 (95% CI: 0.84, 1.17), and the corresponding estimate among users with at least 2 years duration was similar. Our findings suggest that aspirin at cardioprophylactic doses as well as paracetamol at analgesic doses is associated with a reduced risk of breast cancer.

There is mounting epidemiological evidence suggesting that nonsteroidal anti-inflammatory drugs (NSAIDs) may substantially reduce the risk of colorectal cancer (Thun *et al*, 1991; García Rodríguez and Huerta-Álvarez, 2000). The effects of NSAIDs in other cancers have also been extensively studied in the last decade. Nonsteroidal anti-inflammatory drugs have been shown to prevent breast cancer in animal models (Lala *et al*, 1997; Robertson *et al*, 1998). Proposed mechanisms commonly involve the inhibition of cyclooxygenase-2 (COX-2) (Sjodahl, 2001), the enzyme responsible for the production of various prostaglandins that play a key role in the proliferation of tumour tissue; there is accumulating evidence that NSAIDs may have the ability to restore apoptosis and inhibit angiogenesis (Thun *et al*, 2002).

Observational studies of the effect of NSAIDs in breast cancer have shown inconclusive results through a meta-analysis, including data from 15 studies that concluded that NSAIDs could be associated with a small decrease in risk (Khuder and Mutgi, 2001). However, the association between breast cancer incidence and glucocorticoid therapy has been hardly explored. Results from *in vitro* studies suggest that glucocorticoids have a direct inhibitory effect on proliferation of mammary cancer cells (Goya *et al*, 1993). However, to our knowledge, this hypothesis has not been tested in an epidemiological study.

We conducted a cohort study with a nested case−control analysis to evaluate the effect of anti-inflammatory drugs in breast cancer incidence using the General Practice Research Database (GPRD).

## MATERIALS AND METHODS

We used data from the GPRD. This database contains computerised information entered by general practitioners (GPs) in the UK (García Rodríguez and Pérez Gutthann, 1998). Data on over two million patients are systematically recorded and sent anonymously to the Medicines and Healthcare products Regulatory Agency (MHRA), which collects and organises this information in order to be used for research projects. The computerised information includes demographics, details from general practitioner's visits, diagnoses from specialist's referrals and hospital admissions, results of laboratory tests and a free text section. Prescriptions issued by the general practitioner are directly generated from the computer. Several studies with the GPRD have documented the validity and completeness of this database (Jick *et al*, 1991).

### Study population

We identified all female subjects 30–79 years old between January 1995 and December 2001. Women became members of the study population on the first day of the study period when they met the criteria of at least 1 year enrolment with the GP and 1 year since the first computerised prescription. That date was their start date. Study members with a code for cancer before start date were excluded. We also excluded women 70 years and older at start date with a follow-up greater than 1 year and no data recorded during their total follow-up time: an indicator of nonassistance with their GP. Our final study cohort comprised 734 899 women.

### Follow-up

All study members were followed from start date until the earliest occurrence of one of the following end points: recorded diagnosis of breast cancer, any cancer other than breast, age of 80 years, death or end of study period (December 2001).

### Case ascertainment and validation

We identified 4005 patients with a code of breast cancer and manually reviewed their computerised patient profiles. Information included demographic data and all clinical information with no personal identifiers. We excluded 297 women: the main reasons were the computer diagnosis was subsequently not confirmed (60%) and prevalent cases (31%). A previous study validating a large number of cancer cases documented a high reliability of cancer diagnoses recorded in the GPRD (Jick *et al*, 1997). In our study, we sent questionnaires to the GPs for validation of a random sample of 114 cases. A total of 108 (95%) questionnaires were received, and all of them confirmed the computer diagnosis of breast cancer. In the end, 3708 patients were considered incident cases of breast cancer.

### Cohort and nested case–control analysis

All cases of breast cancer (*n*=3708) identified in the study cohort were used in the nested case–control analysis and we considered their date of initial diagnosis as index date. A date during the study period was generated at random for every member of the study cohort. If the random date of a study member was included in her eligible person-time, we used her random date as the index date and marked that woman as an eligible control. This selection mechanism allows that the likelihood of being selected as a control is proportional to the person-time at risk. The same eligibility criteria were applied to controls as to cases. In total, 20 000 controls were frequency-matched by age (interval of one year) and calendar year from the list of all eligible controls.

Estimates of odds ratio (OR), assumed to be valid estimates of the relative risk, and 95 percent confidence interval (95% CI) associated with use of aspirin, non-aspirin NSAIDs, paracetamol, and oral steroids compared to nonuse were computed using unconditional logistic regression. We ascertained patients with previous benign breast disease recorded at least more than 1 year before the index date. Other risk factors like alcohol intake, body mass index (BMI) and hormone replacement therapy (HRT) were also ascertained based on their medical history recorded in the GPRD. We also elicited subjects’ use of health services (visits to the GP, specialist referrals and hospital admissions) in the 2 years prior to the index date. All estimates of OR were adjusted for age, calendar year, BMI, alcohol intake, smoking status, HRT use and prior benign breast disease.

### Exposure definition

We defined three time windows of exposure for aspirin, non-aspirin NSAIDs, paracetamol and oral steroids: current use, past use and nonuse. Current use was defined as use that lasted until the index date or ended in the year prior to the index date based on the supply of drug therapy as prescribed by the GP. Past use was use that ended more than 1 year before the index date. Finally, the time window of nonuse was defined as no recorded use at any time before the index date. Current users were subdivided into short-term and long-term users. Short-term users were patients whose duration of treatment was shorter than 1 year and long-term users were patients treated for a period longer than 1 year.

The effect of dose was also analysed among long-term users. Aspirin daily dose was assigned into three groups: 75, 150 and 300 mg. Specific cutoff values for each individual non-aspirin NSAID were used to group non-aspirin NSAID daily dose into two categories; low-medium and high dose. Daily doses of paracetamol up to 1000 mg were assigned into a low-dose category, doses above 1000 mg and up to 2000 mg were assigned into a medium dose category, while doses above 2000 mg were grouped into a high-dose category. Oral steroid daily doses up to 10 mg of prednisolone (or equivalent dose for other steroids) were classified as low-medium, while daily doses of 10 mg and above were classified as high. An additional analysis using 2 years time lag (advancing the index date by 2 years in cases and controls) was performed.

## RESULTS

The incidence rate of breast cancer in our study population was 156 per 100 000 person-years among women 30–79 years old, well in line with other reports from the UK (Office for National Statistics, 2002).

Aspirin was associated with a decreased risk of breast cancer (Table 1

**Table 1:** Risk of breast cancer associated with aspirin use

	**Cases (*n*=3708)**	**Controls (*n*=20 000)**	**Odds ratio^a^ (95% CI)**
*Aspirin use*			
No use^b^	3420	18 260	
Current use	205	1241	0.88 (0.75, 1.04)
Past use	83	499	0.90 (0.70, 1.15)
			
*Aspirin duration*			
0–0.9 years	88	438	1.11 (0.87, 1.41)
1–1.9 years	37	245	0.78 (0.54, 1.13)
2–3.9 years	34	274	0.66 (0.45, 0.96)
4+ years	46	284	0.86 (0.61, 1.19)
			
*Aspirin dose (mg)*^c^			
75	63	498	0.67 (0.51, 0.89)
150	34	185	0.96 (0.65, 1.41)
300	20	120	0.89 (0.54, 1.46)

a Adjusted for age, calendar year, BMI, smoking, alcohol, prior benign breast disease, NSAIDs, paracetamol, steroid and HRT use.

b Reference category.

c Among current long-term users (1 or more years of treatment duration) *vs* nonusers.

). Women taking aspirin for 1 year or longer had an OR of 0.77 (95% CI: 0.62, 0.95) compared to nonusers. The observed effect was stronger among women using 75 mg daily. The analysis using 2 years lag time yielded similar results and current users of aspirin for 1 year or longer had an OR of 0.81 (95% CI: 0.62, 1.06) compared to nonusers (Table 2

**Table 2:** Risk of breast cancer associated with aspirin, non-aspirin nonsteroidal anti-inflammatory drugs (NSAID), paracetamol and steroid use duration using the 2 years lag-time analysis

	**Cases (*n*=3708)**	**Controls (*n*=20 000)**	**Odds ratio^a^ (95% CI)**
*Aspirin*			
No use^b^	3524	18 866	
Current use	122	792	0.84 (0.69, 1.02)
0–0.9 years	57	349	0.87 (0.66, 1.16)
1–1.9 years	15	124	0.65 (0.38, 1.12)
2–3.9 years	31	193	0.88 (0.60, 1.30)
4+ years	19	126	0.87 (0.53, 1.41)
Past use	62	342	0.96 (0.73, 1.26)
			
*Non-aspirin NSAIDs*			
No use^b^	1884	10 138	
Current use	802	4444	0.98 (0.89, 1.08)
0–0.9 years	606	3346	0.98 (0.88, 1.09)
1–1.9 years	62	360	0.93 (0.70, 1.23)
2–3.9 years	67	377	0.97 (0.74, 1.28)
4+ years	67	361	1.05 (0.80, 1.38)
Past use	1022	5418	1.01 (0.93, 1.11)
			
*Paracetamol*			
No use^b^	2244	11 762	
Current use	744	4345	0.88 (0.79, 0.97)
0–0.9 years	499	2760	0.92 (0.83, 1.03)
1–1.9 years	70	393	0.92 (0.70, 1.19)
2–3.9 years	87	606	0.72 (0.57, 0.92)
4+ years	88	586	0.76 (0.60, 0.97)
Past use	720	3893	0.95 (0.86, 1.04)
			
*Oral steroids*			
No use^b^	3452	18 567	
Current use	124	704	0.96 (0.79, 1.17)
0–0.9 year	96	484	1.08 (0.87, 1.35)
1–1.9 years	12	60	1.04 (0.56, 1.95)
2–3.9 years	11	70	0.93 (0.49, 1.76)
4+ years	5	90	0.31 (0.12, 0.76)
Past use	132	729	0.98 (0.81, 1.19)

a Estimates are adjusted for age, calendar year, BMI, alcohol intake, smoking status, HRT use, prior benign breast disease, and all the variables in the table using logistic regression.

b Reference category.

).

Non-aspirin NSAID use was not associated with a reduced risk of breast cancer (Table 3

**Table 3:** Risk of breast cancer associated with non-aspirin NSAID use

	**Cases (*n*=3708)**	**Controls (*n*=20 000)**	**Odds ratio^a^ (95% CI)**
*Non-aspirin NSAID use*			
No use^b^	1563	8398	
Current use	852	4465	0.98 (0.88, 1.09)
Past use	1293	7137	0.94 (0.86, 1.03)
			
*Non-aspirin NSAID duration*			
0–0.9 years	611	3208	0.98 (0.87, 1.09)
1–1.9 years	84	387	1.13 (0.87, 1.47)
2–3.9 years	64	357	0.92 (0.69, 1.23)
4+ years	93	513	0.94 (0.74, 1.21)
			
*Non-aspirin NSAID dose*^c^			
Low-medium	128	658	0.99 (0.80, 1.23)
High	113	599	0.99 (0.79, 1.24)
			
*Non-aspirin NSAID indication*^c^			
OA	207	1063	0.98 (0.82, 1.17)
RA	26	145	0.96 (0.61, 1.51)
Other pain	8	49	0.84 (0.38, 1.86)

OA=osteoarthritis, RA=rheumatoid arthritis; NSAID=nonsteroidal anti-inflammatory drugs.

a Adjusted for age, calendar year, BMI, smoking, alcohol, prior benign breast disease, paracetamol, aspirin, steroid and HRT use.

b Reference category.

c Among current long term users (1 or more years of treatment duration) *vs* non-users. Cut-off values for dose in mg were: aceclofenac 100, acemetacin 120, apazone 600, diclofenac 100, etodolac 400, fenbufen 900, fenoprofen 1200, flurbiprofen 150, ibuprofen 1200, indomethacin 75, ketoprofen 150, mefenamic 1000, meloxicam 7.5, nabumetone 1000, naproxen 750, piroxicam 10, sulindac 200, tenoxicam 10, and tiaprofenic acid 450.

). The estimate of risk among Non-aspirin NSAID users with treatment duration longer than 1 year was 1.00 (95% CI: 0.84, 1.17). Among these women, the risk was no different between dose groups or treatment indication groups. non-aspirin NSAID users with at least 2 years duration presented an OR of 0.93 (95% CI: 0.76, 1.13). All these results were similar to those observed with the 2 years lag time analysis (Table 2).

We found that paracetamol was associated with a decreased risk of breast cancer (Table 4

**Table 4:** Risk of breast cancer associated with paracetamol use

	**Cases (*n*=3708)**	**Controls (*n*=20 000)**	**Odds ratio^a^ (95% CI)**
*Paracetamol use*			
No use^b^	1885	10 040	
Current use	878	4943	0.90 (0.82, 1.00)
Past use	945	5017	0.97 (0.89, 1.07)
			
*Paracetamol duration*			
0–0.9 years	589	2 949	1.00 (0.89, 1.12)
1–1.9 years	57	489	0.63 (0.47, 0.84)
2–3.9 years	83	531	0.81 (0.63, 1.05)
4+ years	149	974	0.77 (0.64, 0.94)
			
*Paracetamol dose*^c^			
Up to 1 g	120	886	0.68 (0.55, 0.84)
Up to 2 g	105	785	0.73 (0.58, 0.91)
More than 2 g	64	323	1.06 (0.79, 1.41)
			
*Paracetamol indication*^c^			
OA	241	1 649	0.76 (0.64, 0.89)
RA	17	112	0.78 (0.45, 1.35)
Other pain	31	233	0.70 (0.47, 1.05)
			
*Paracetamol preparation*^c^			
Paracetamol	48	354	0.69 (0.49, 0.95)
Paracetamol with codeine^d^	128	817	0.83 (0.67, 1.02)
Paracetamol with propoxyphene	111	799	0.71 (0.57, 0.89)

OA=osteoarthritis, RA=rheumatoid arthritis.

a Adjusted for age, calendar year, BMI, smoking, alcohol, prior benign breast disease, NSAIDs, aspirin, steroid and HRT use.

b Reference category.

c Among current long-term users (1 or more years of treatment duration) *vs* nonusers.

d Includes both codeine and dihydrocodeine.

). The OR among women treated with paracetamol for 1 year or longer was 0.76 (95% CI: 0.65, 0.88) compared to nonusers. This reduced risk was observed among low-dose and medium-dose users (OR=0.68, 95% CI: 0.55, 0.84, and OR=0.73, 95% CI: 0.58, 0.91) but not among high-dose users (OR=1.06, 95% CI: 0.79, 1.41). The protective effect was observed independently of the treatment indication. We also found the effect to be similar irrespective of whether paracetamol was used alone or in combinations. The 2 years lag time analysis provided similar results (Table 2).

Women taking oral steroids during 1 year or longer had an OR of 0.85 (95% CI: 0.60, 1.21) compared to nonusers. Women with the longest duration of use (4 years or more) presented a lower risk (OR=0.66, 95% CI: 0.36, 1.18) (Table 5

**Table 5:** Risk of breast cancer associated with oral steroid use

	**Cases (*n*=3708)**	**Controls (*n*=20 000)**	**Odds ratio^a^ (95% CI)**
*Oral steroid use*			
No use^b^	3370	18 090	
Current use	132	820	0.83 (0.68, 1.02)
Past use	206	1090	0.99 (0.84, 1.16)
			
*Oral steroid duration*			
0–0.9 years	93	560	0.84 (0.66, 1.07)
1–1.9 years	9	59	0.88 (0.42, 1.80)
2–3.9 years	17	82	1.09 (0.63, 1.90)
4+ years	13	119	0.66 (0.36, 1.18)
			
*Oral steroid dose*^c^			
Low-medium	34	212	0.90 (0.62, 1.32)
High	5	48	0.57 (0.22, 1.47)
			
*Oral steroid indication*^c^			
Respiratory disease	13	70	1.13 (0.61, 2.08)
Osteoarthritis	4	28	0.69 (0.23, 2.09)
Polymyalgia rheumatica	8	57	0.77 (0.36, 1.65)
Rheumatoid arthritis	9	57	0.96 (0.46, 1.99)
Other	5	48	0.53 (0.20, 1.38)

a Estimates are adjusted for age, calendar year, BMI, alcohol intake, smoking status, HRT, aspirin, NSAID, paracetamol, and prior benign breast disease using logistic regression.

b Reference category.

c Among current long-term users (1 or more years of treatment duration) *vs* nonusers.

). Small numbers were available to study the effect among long-term users by treatment indication and dose, but we found the reduced risk to be more apparent among high-dose users of steroids. The 2 years lag time analysis did not affect the results (Table 2).

We studied the effect of aspirin, non-aspirin NSAIDs, paracetamol and oral steroids among women aged 55 years or older and below 55 separately. The results in both age groups were similar to the ones observed overall (data not shown).

## DISCUSSION

We found that use of aspirin and paracetamol was associated with a reduced risk of breast cancer of about 20%. On the other hand, we found little evidence for a protective effect of non-aspirin NSAIDs. These results are quite similar to those found in a recent study performed with an identical design and using the same source population that addressed the risk of another hormone-dependent cancer in men, in this case prostate cancer (García Rodríguez and González-Pérez, 2004).

We identified 10 previous studies evaluating the association between aspirin and breast cancer. Among them, eight reported an estimate in line with our observed 20% risk reduction (Thun *et al*, 1993; Schreinemachers and Everson, 1994; Harris *et al*, 1996; Neugut *et al*, 1998; Coogan *et al*, 1999; Harris *et al*, 1999; Cotterchio *et al*, 2001; Johnson *et al*, 2002). The other two studies found no effect (Egan *et al*, 1996; Anderson *et al*, 2002). The summary estimate for these studies is 0.78 (95% CI: 0.70, 0.86) (González-Pérez *et al*, 2003).

As opposed to aspirin, long-term use of non-aspirin NSAIDs was not associated with a reduced risk of developing breast cancer in our study. Among the four studies that previously reported an estimate for non-aspirin NSAIDs, two of them found a negative result (Egan *et al*, 1996; Johnson *et al*, 2002) and the other two studies found a suggestion of a slight risk reduction (Coogan *et al*, 1999; Cotterchio *et al*, 2001). The summary estimate for these studies is 0.89 (0.79–1.01) (González-Pérez *et al*, 2003). In our study, the indication among long-term users of aspirin was predominantly cardiovascular prevention. When we restricted the analysis to women with cardiovascular comorbidity, we did not see either any suggestion of a reduced risk among users of non-aspirin NSAIDs (data not shown). It must be noted that in our study recorded use of non-aspirin NSAIDs of more than 5 years was rare. Therefore, we could not assess the effect of very extended durations of non-aspirin NSAIDs on breast cancer occurrence with confidence.

A reduced risk among paracetamol users has been previously reported in two studies. Meier *et al* (2002)found that those with 30 paracetamol prescriptions or more had an OR of 0.8 (95% CI: 0.7, 1.0) compared to nonusers. Harris *et al* (1999) reported an OR of 0.84 among paracetamol users in a cohort of 32 505 women. Additionally, paracetamol has been previously linked to a decreased risk of ovarian cancer, another hormone-related cancer (Cramer *et al*, 1998; Rodriguez *et al*, 1998; Friis *et al*, 2002). In our study, we observed that users of paracetamol presented a reduced risk of breast cancer. However, this effect was restricted to users taking no more than 2000 mg daily, whereas users of higher doses did not present this risk reduction. A similar pattern of dose was observed when we looked at the effect stratified by different treatment indications (data not shown). We found that paracetamol either in single or combined preparations shared the same effect.

Previous *in vitro* studies have shown that glucocorticoids have a direct inhibitory effect on proliferation of mammary cancer cells (Goya *et al*, 1993). The biological mechanism is not well understood. In fact, in normal breast tissue, glucocorticoids have been reported to induce the synthesis of aromatase (Chen, 1998): an enzyme that catalyses the conversion of androgens to oestrogens, and is the target site for a new class of breast cancer chemotherapy. However, this effect has not been observed in cancerigenous tissue. Our results suggest that women taking oral steroid therapy for extended duration could present a decreased risk of breast cancer, although it should be noted that the confidence intervals were large.

The present study has some limitations. First of all, information on drug exposure came from prescriptions written by GPs. Over-the-counter (OTC) use of NSAIDs, paracetamol or aspirin is not recorded on computer files. However, the proportion of OTC use (preferentially short-term treatment) out of the total use of chronic treatment is small. Yet, nondifferential misclassification of drug use due to noncompliance or OTC use would tend to attenuate the true association between the specific drug group and breast cancer (Ulcickas Yood *et al*, 2000). We could indirectly assess the magnitude of the misclassification using data from a study in the UK, in which subjects were interviewed to obtain information on use of prescribed and self-medication in the last 3 months (Langman *et al*, 1994). In order to be able to compare meaningfully both studies, one must take into account the differences in the study populations. Their study, that included both males and females, had 25% of the controls over 80 years old, whereas our study included only females up to 79 years of age. Prevalence of non-aspirin NSAID use in their study was 18% in the control series, compared to 12% in our study using a similar time window. Similarly, prevalence of paracetamol use was 20% in their study, whereas in our study it was 16%. Since the use of most medications (including NSAIDs and paracetamol) is greater among elderly, it is likely that no major under-recording of long-term NSAID or paracetamol use was present in our data after allowing for the different age distribution. Finally, the prevalence of aspirin was 18% in their study compared with 6% in our study. Since our population included only women whereas theirs included both men and women, we do expect a significantly lower prevalence of chronic aspirin use (mainly used in cardioprophylaxis). Indeed, in a previous study of prostate cancer using the same database where only males were included, we observed a prevalence of aspirin use close to 17%. Therefore, we can also conclude that most likely no major under-recording of long-term aspirin use was present in our data after allowing for the different sex and age distributions.

In some instances, a breast cancer diagnosis might be preceded by pain symptoms and/or a greater use of health services. This could translate into a spurious greater exposure to pain medications among cases than controls. In order to overcome this potential bias, we performed a 2 years lag time analysis advancing the index date by 2 years in cases and controls. In this secondary analysis, virtually all estimates of effect from the main analysis were replicated. Also, adjusting for health services utilisation (visits to GPs, specialist referrals, hospital admissions) did not materially change the results (data not shown).

Although we recorded information regarding traditional risk factors for breast cancer including age, alcohol use, smoking, BMI, HRT use and previous breast abnormalities, there is still room for some confounding from either measurement bias or other risk factors that we were not able to elicit such as age at menarche, parity, family history, age at first child or germline mutation. However, we would expect these risk factors to be rather evenly distributed among users and nonusers of the study drugs and consequently affecting very little, if any, our estimates of effect.

In summary, our findings suggest that use of aspirin at cardioprophylactic doses is associated with a reduced risk of breast cancer, but there was little evidence for a reduced risk among users of non-aspirin NSAIDs. We also found long-term use of paracetamol to be linked to a reduced risk of breast cancer.
